# Supplementary material for: Enhanced Electrochemical Water Oxidation Activity by Structural Engineered Prussian Blue Analogue/rGO Heterostructure
Source: Molecules. 2022 Aug 25;27(17):5472. doi: 10.3390/molecules27175472 (PMC9458107; doi:10.3390/molecules27175472)
Supplement: Supplementary file 1 [file molecules-27-05472-s001.zip › molecules-1864268-supplementary.pdf]

Supporting Information

Enhanced Electrochemical Water Oxidation Activity by Structural Engineered Prussian Blue Analogue/rGO Heterostructure

Xiuyun An [1], Weili Zhu [1], Chunjuan Tang [1], Lina Liu [1], Tianwei Chen [2], Xiaohu Wang [2], Jianguo Zhao [3,*] and Guanhua Zhang [2,*]

[1] Department of Mathematics and Physics, Luoyang Institute of Science and Technology, Luoyang 471023, China

[2] State Key Laboratory of Advanced Design and Manufacturing for Vehicle Body, College of Mechanical and Vehicle Engineering, Hunan University, Changsha, 410082, China

[3] College of Physical & Electronic Information，Luoyang Normal University, Luoyang 471934, China

[*] Corresponding authors: 2jg_1981@163.com (J.Z.); guanhuazhang@hnu.edu.cn (G. Z.)

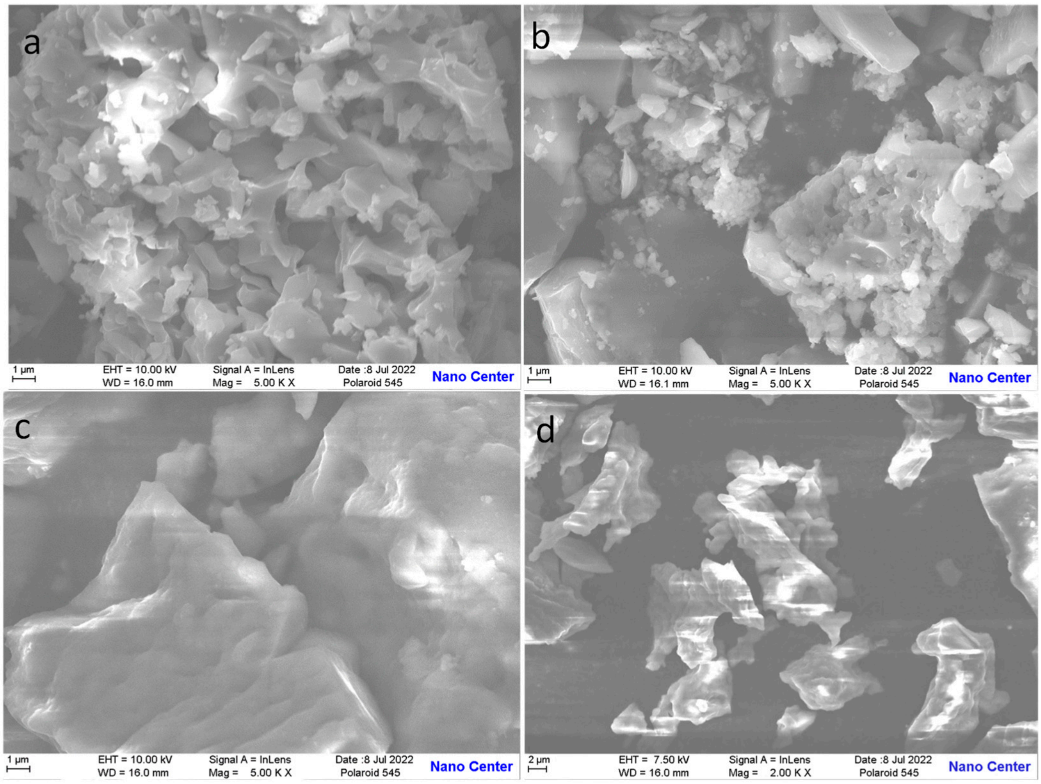

Figure S1. SEM images of as-obtained PBA materials. (**a**) PBA 0-1; (**b**) PBA 1-1; (**c**) PBA 3-1; (**d**) PBA 4-1.

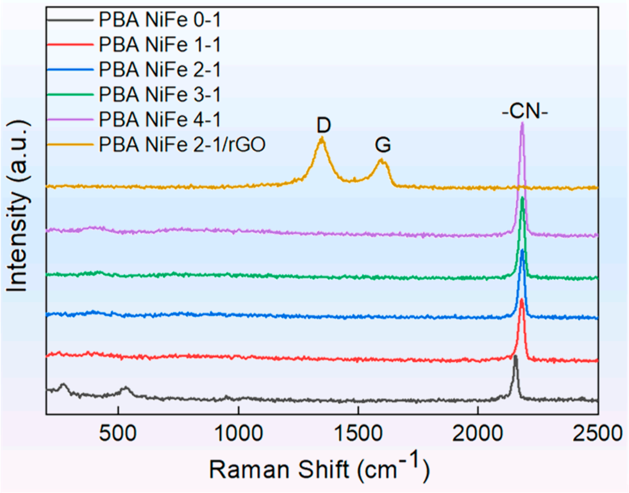

Figure S2. The Raman spectra of PBA materials.

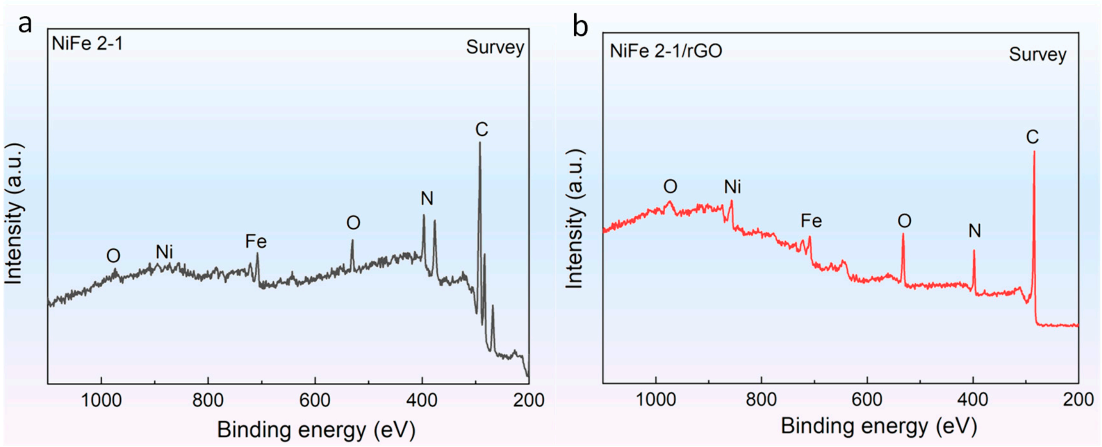

Figure S3. The XPS survey spectra of PBA materials before OER tests. (**a**) PBA NiFe 2-1; (**b**) PBA NiFe 2-1/rGO.

Table S1. Catalyst performance of analogous materials.

Materials	Methods	Mass loading /mg cm^{-2}	Overpotential /mV (30 $mAcm^{-2}$)	Tafel slopes /mV dec^{-1}	Durability /h	Ref
NiHCF-200c	Sodium-ion electrochemical tuning method	0.5	300	41	24	[1]
FeCoNi PBAs polyhedrons	Oil bath reaction	0.5	300	52	–	[2]
Ni-Fe-$K_{0.23}MnO_2$ CNFs-300	Sacrificial template strategy	2	290	42.3	24	[3]
NiCo@A-NiCo-PBA-AA	Topological growth method	0.0006 (GCE)	410	79.1	40	[4]
(v-NiFe PBA@rGO	Rapid coprecipitation method	0.285	290	36.2	200	[5]
PBA NiFe 2-1/rGO	Coprecipitation method	0.5	331.5	57.9	40	This work

GCE: glassy carbon electrode.

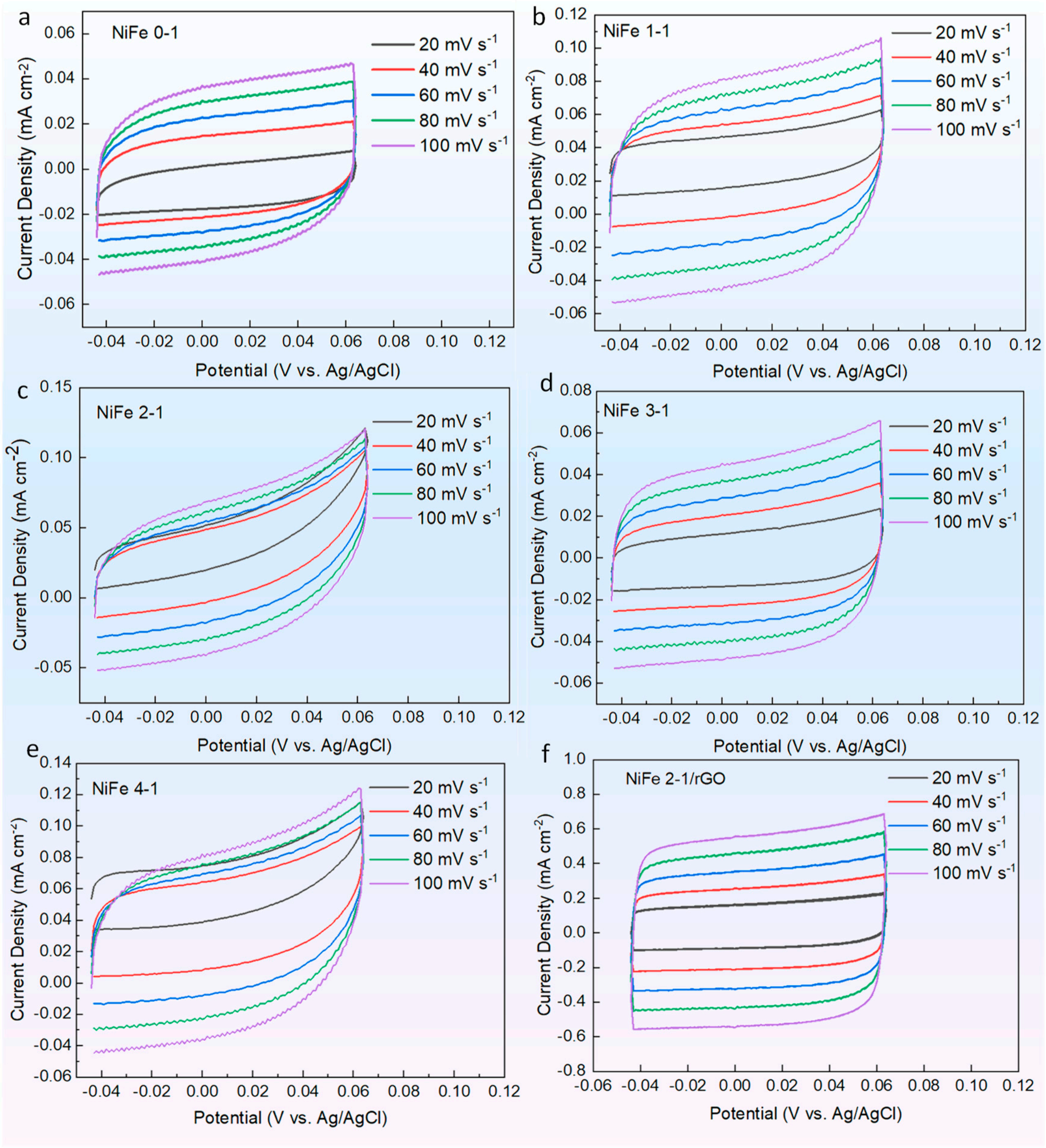

Figure S4. CV curves of as-obtained PBA materials at different scan rates in non-Faraday potential range. (**a**) PBA NiFe 0-1; (**b**) PBA NiFe 1-1; (**c**) PBA NiFe 2-1; (**d**) PBA NiFe 3-1; (**e**) PBA NiFe 4-1; (**f**) PBA NiFe 2-1/rGO.

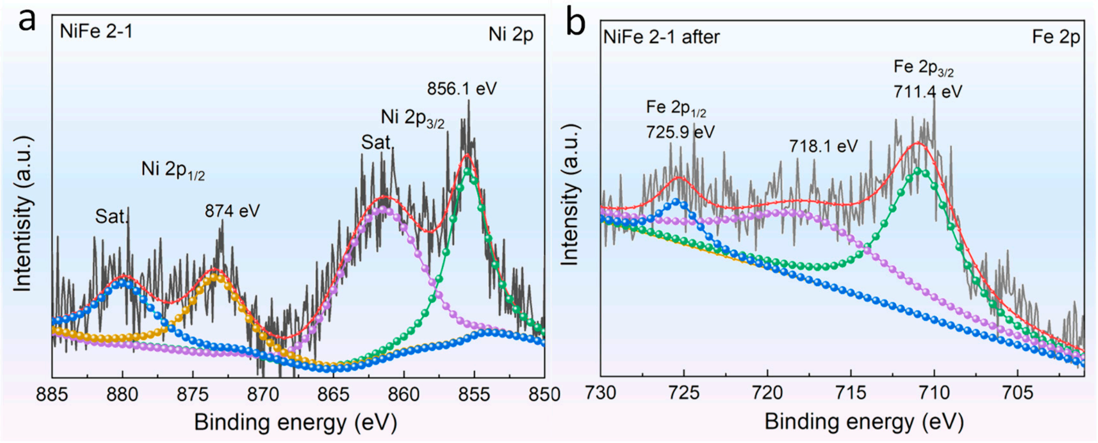

Figure S5. XPS spectra of PBA samples after OER measurement. High-resolution Ni 2p (**a**) and Fe 2p (**b**) spectra of PBA NiFe 2-1, respectively.

Table S2. The calculated Cdl ECSA values of as-obtained PBA materials.

Materials	C_{dl} (mF cm^{-2})	ECSA (cm^2)
PBA NiFe 0-1	0.4	10
PBA NiFe 1-1	0.6	15
PBA NiFe 2-1	0.5	12.5
PBA NiFe 3-1	0.4	10
PBA NiFe 4-1	0.5	12.5
PBA NiFe 2-1/rGO	5.3	132.5

References

1. Tang, S.; Li, L.; Ren, H.; Lv, Q.; Lv, R., Sodium-ion electrochemical tuning of Prussian blue analog as an efficient oxygen evolution catalyst. *Mater. Today Chem.* **2019,** 12, 71-77.

2. Du, Y.; Ding, X.; Han, M.; Zhu, M., Morphology and Composition Regulation of FeCoNi Prussian Blue Analogues to Advance in the Catalytic Performances of the Derivative Ternary Transition-Metal Phosphides for OER. *ChemCatChem* **2020,** 12, 4339-4345.

3. Liao, H.; Guo, X.; Hou, Y.; Liang, H.; Zhou, Z.; Yang, H., Construction of Defect-Rich Ni-Fe-Doped K0.23MnO2 Cubic Nanoflowers via Etching Prussian Blue Analogue for Efficient Overall Water Splitting. *Small* **2020,** 16, 1905223.

4. Zhang, H.; Li, P.; Chen, S.; Xie, F.; Riley, D. J., Anodic Transformation of a Core-Shell Prussian Blue Analogue to a Bifunctional Electrocatalyst for Water Splitting. *Adv. Funct.l Mater.* **2021,** 31, 2106835.

5. Jo, S.; Kwon, J.; Choi, S.; Lu, T.; Byeun, Y.; Han, H.; Song, T., Engineering $[Fe(CN)_6]_3^-$ vacancy via free-chelating agents in Prussian blue analogues on reduced graphene oxide for efficient oxygen evolution reaction. *Appl. Surf. Sci.* **2022,** 574, 151620.
